# Supplementary material for: Multisite phosphorylation drives phenotypic variation in (p)ppGpp synthetase-dependent antibiotic tolerance
Source: Nat Commun. 2019 Nov 13;10:5133. doi: 10.1038/s41467-019-13127-z (PMC6853874; doi:10.1038/s41467-019-13127-z)
Supplement: Supplementary file 1 — Supplementary Information [file 41467_2019_13127_MOESM1_ESM.pdf]

Supplementary Information for

Multisite phosphorylation drives phenotypic variation in (p)ppGpp synthetase-dependent antibiotic tolerance

Libby et al.

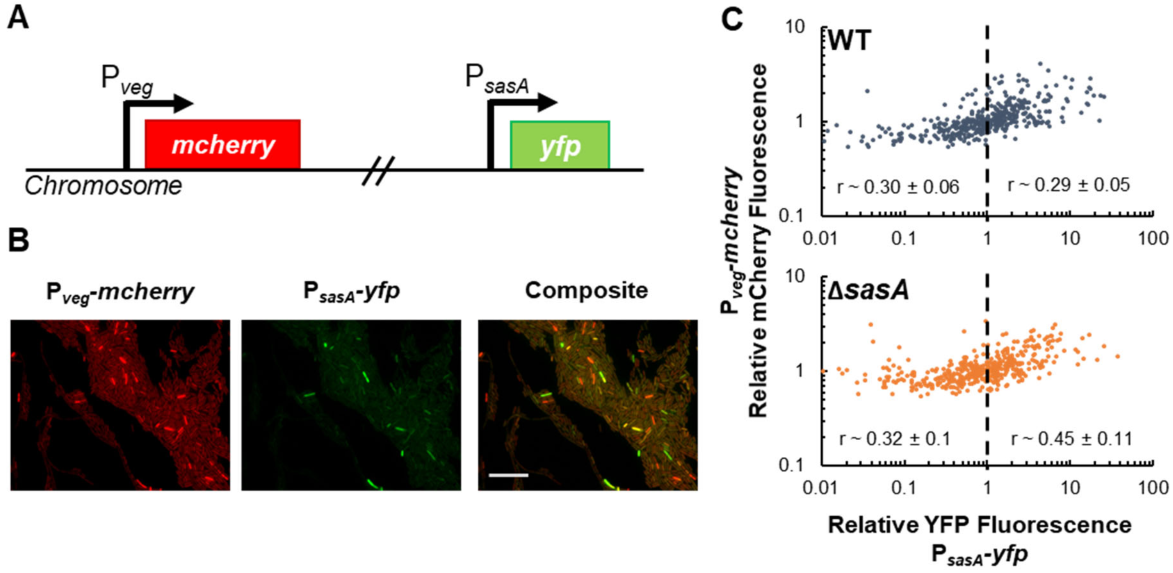

Supplementary Figure 1: Comparison of P$_{sasA}$-*yfp* and the constitutive reporter P$_{veg}$-*mcherry*

A) Schematic of dual-color transcriptional reporter strain used to measure the correlation in the expression of *sasA* and *veg*. Two transcriptional reporters, P$_{veg}$-*mcherry* and P$_{sasA}$-*yfp* are inserted at ectopic loci.

B) Correlation of P$_{veg}$ and P$_{sasA}$ activity in single cells. Individual fluorescence channels and a composite image of a dual reporter strain P$_{sasA}$-*yfp* P$_{veg}$-*mcherry* in log phase growth. **Left:** P$_{veg}$-*mcherry* reporter displays cell-to-cell variability. **Center:** P$_{sasA}$-*yfp* reporter in the same population displays cell-to-cell variability. **Right:** Composite image with mCherry and YFP images. Scale bar indicates 10 μm.

C) Quantification of the correlation between P$_{sasA}$-*yfp* and P$_{veg}$-*mcherry* expression in single cells in an otherwise WT (**top**) or a Δ*sasA* (**bottom**) background. Cellular fluorescence intensities were measured for single cells described in **(A,B)** in 4 experiments (at least 540 cells). Relative fluorescence is the fluorescence of each individual cell relative to the mean fluorescence of the population in each channel and is plotted on a linear scale

(mCherry) or a log scale (YFP). The Pearson's correlation coefficients above and below mean *sasA* expression (relative fluorescence = 1) are indicated on the graphs (mean ± SEM, 4 experiments). The correlation between P_{sasA}-*yfp* and P_{veg}-*mcherry* is not robustly stronger in cells with above mean *sasA* expression. Over the entire range, the Pearson's correlation coefficients are: WT ~ 0.48 ± 0.01, Δ*sasA* ~ 0.51 ± 0.10; the Spearman correlation coefficients are: WT ~ 0.58 ± 0.05, Δ*sasA* ~ 0.55 ± 0.04. The CV of P_{veg} is 0.40 ± 0.02 (mean ± SEM, 8 experiments).

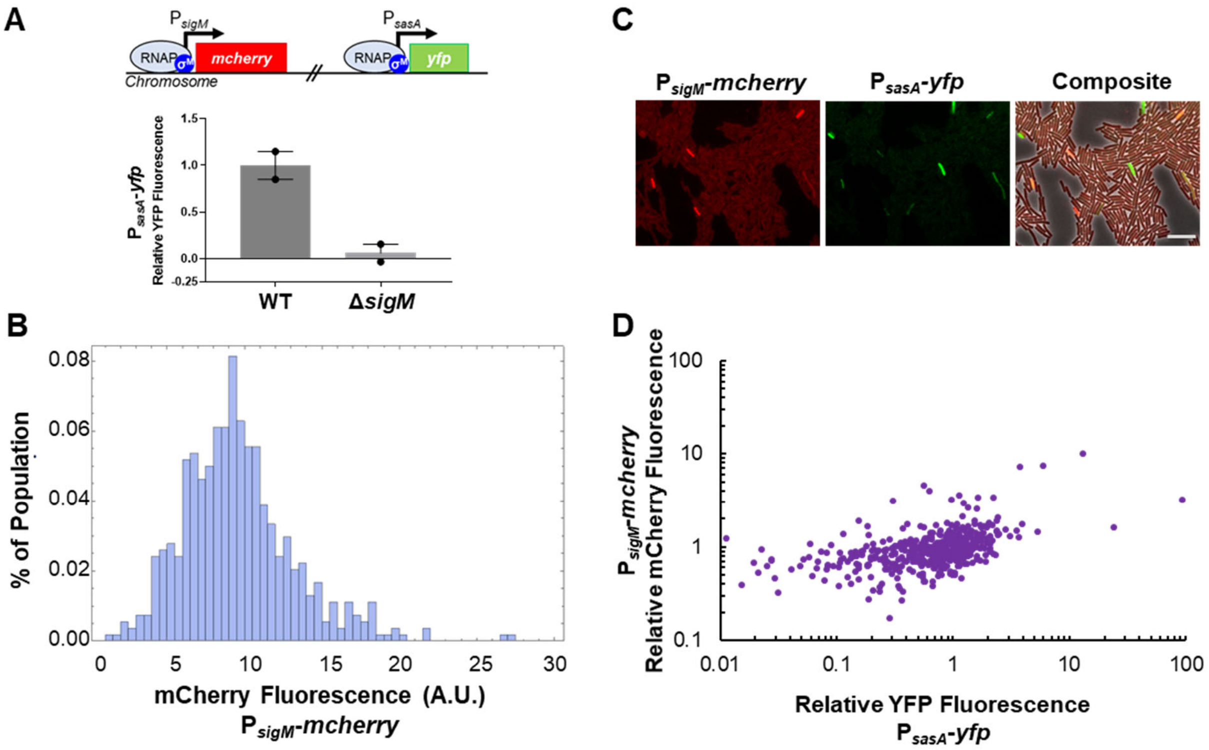

Supplementary Figure 2: Correlation of cell-to-cell variability in *sigM* and *sasA*

A) Top: Schematic of dual-color transcriptional reporter strain used to measure the correlation in the expression in *sasA* and *sigM*. Two transcriptional reporters, P_{sigM}-*mcherry* and P_{sasA}-*yfp,* are inserted at ectopic loci. Both *sigM* and *sasA* transcription use the alternative sigma factor σ^M (SigM) of RNAP. Bottom: In a Δ*sigM* background, P_{sasA}-*yfp* is not detected (plotted are the mean and range of two experiments, at least 190 cells). Bars and lines indicate the means and SEMs, respectively.

B) Quantification of the distribution of P_{sigM}-*mcherry* expression in a log phase culture in the absence of a specific inducing stress. Histogram of the mCherry fluorescence intensities per cell, background subtracted, for ~540 individual cells.

C) Correlation of P_{sigM} and P_{sasA} activity in single cells. Fluorescence and composite phase contrast images of the dual reporter strain P_{sasA}-*yfp* P_{sigM}-*mcherry* in log phase growth. **Left:** P_{sigM}-*mcherry* reporter displays cell-to-cell variability. **Center:** P_{sasA}-*yfp* reporter in

the same population displays cell-to-cell variability. **Right:** Composite image with phase contrast, mCherry, and YFP images. Scale bar indicates 10 μm.

D) Quantification of the correlation between P_{sasA}-*yfp* and P_{sigM}-*mcherry* expression in single cells. Cellular fluorescence intensities were measured for single cells described in (**A,C)**. The Pearson's correlation coefficient of the relative fluorescence values is r ~ 0.35 ± 0.09, the Spearman's correlation coefficient is 0.53 ± 0.05 (SEM, 3 experiments).

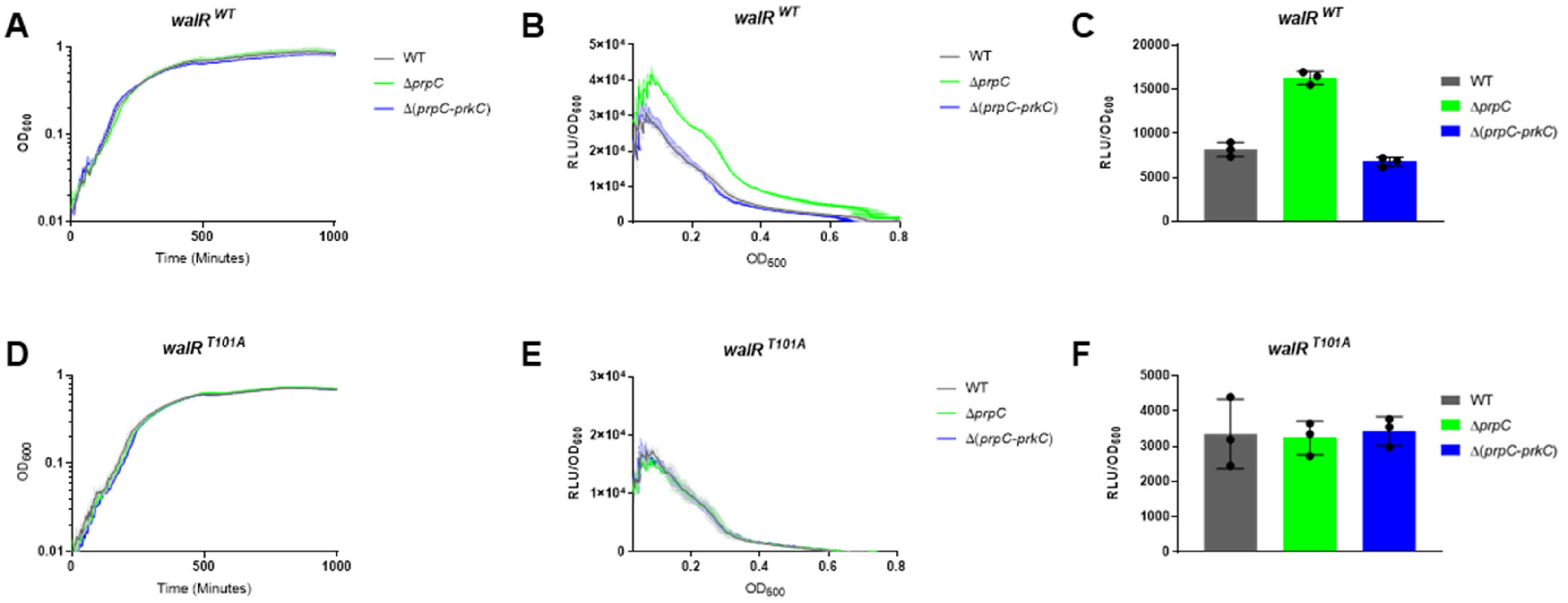

Supplementary Figure 3: PrkC regulates *yocH* during log phase growth in minimal media.

A) Growth curves of P_{yocH}-*luxABCDE* in WT (gray), Δ*prpC* (green), and Δ(*prpC-prkC*) (dark blue) in an otherwise wild type background in the chemically defined minimal medium S7-glucose. Time indicates minutes post OD_{600} ~0.01 for each genotype. For all graphs solid lines indicate the means of 3 experimental replicates, and the shading indicates the standard deviation.

B) Relative luminescence units (RLU) / OD_{600} as a function of OD_{600} for the experiment shown in **A**. Throughout log phase, the normalized reporter activity in the Δ*prpC* background is significantly higher than WT or a Δ(*prpC-prkC*) background.

C) Detail of relative P_{yocH}-*luxABCDE* reporter activity (RLU/OD_{600}) at OD_{600}~0.3.

D-F) A similar experiment to **A-C** was performed in a *walR*^{T101A} background.

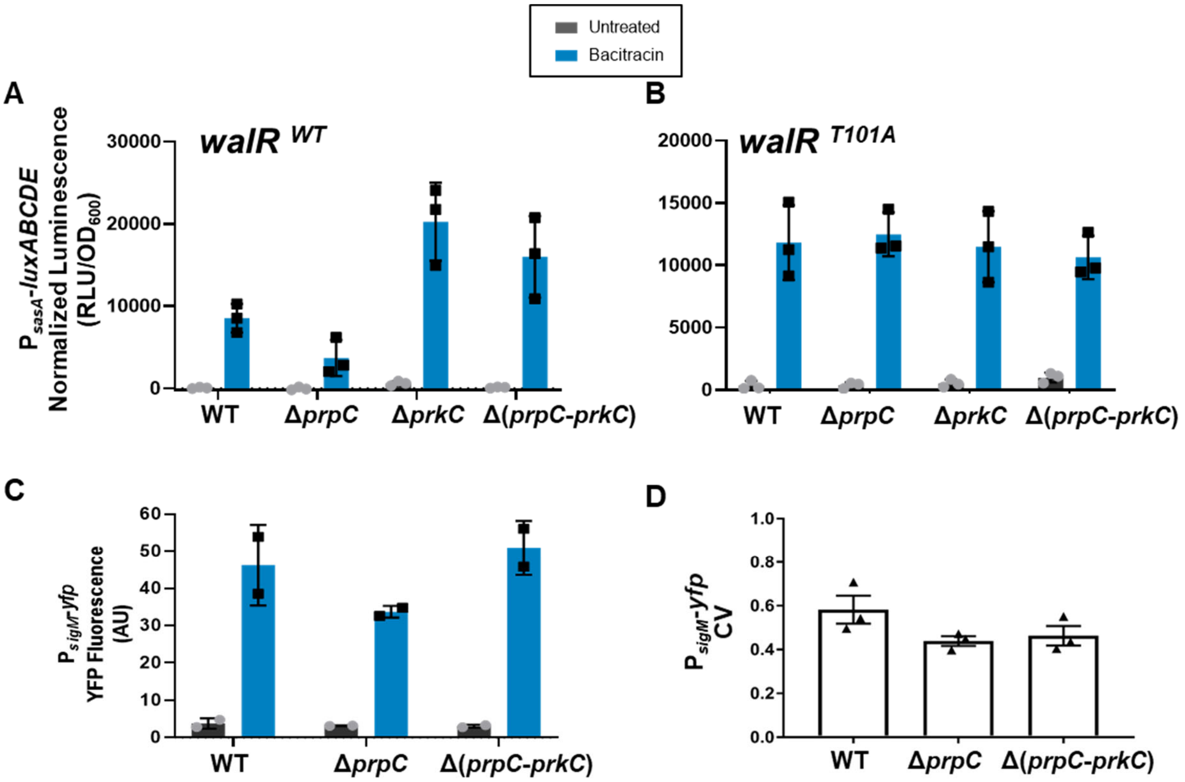

Supplementary Figure 4: The Ser/Thr kinase PrkC regulates average *sasA* induction through WalR T101~P.

A) The Ser/Thr kinase PrkC and its partner phosphatase PrpC regulate *sasA* under inducing conditions. In the absence of induction, *sasA* expression is very low (gray). Bacitracin (blue) induces *sasA* expression amplifying relative changes.

The average induction of *sasA* without (gray) and with bacitracin treatment (blue) was measured in otherwise WT, Δ*prpC*, Δ*prkC*, or Δ(*prpC*-*prkC*) backgrounds using a P_{sasA}-*lux* reporter. A >4 fold change is observed between the Δ*prpC* and Δ(*prpC*-*prkC*) backgrounds, indicating that PrkC activity represses *sasA*. Bars and lines indicate the mean and standard deviation, respectively, of at least 3 biological replicates.

B) PrkC-dependent *sasA* regulation is abrogated in a non-phosphorylatable *walR*^{T101A} mutant background. The average relative induction of *sasA* without (gray) and with bacitracin treatment (blue) was measured as in (**A**), but in a *walR*^{T101A} mutant background. No significant difference between genetic backgrounds is observed.

C) A P_{sigM}-*yfp* reporter was used to measure *sigM* induction during bacitracin treatment in WT, Δ*prpC*, and Δ(*prpC-prkC*) backgrounds. Cells were grown to early log phase and treated with 100 μg/ml bacitracin for ~2h prior to measurement by microscopy. Data represents the average cellular YFP fluorescence of ~200 cells per condition and the bars represent the range in means observed between 2 biological replicates. P_{sigM}-*yfp* expression is ~similar in all genetic backgrounds with or without bacitracin treatment.

D) CVs of a P_{sigM}-*yfp* reporter under non-inducing conditions in WT, Δ*prpC*, and Δ(*prpC-prkC*) backgrounds. Shown are the mean and SEM of the CV (bars and lines) of 3 experiments (triangles); each experiment measured >100 cells.

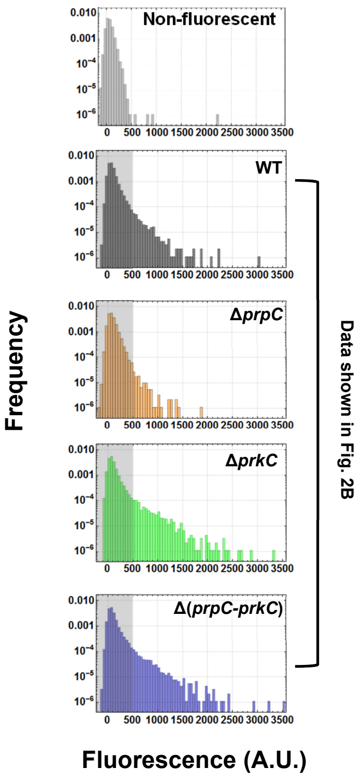

Supplementary Figure 5: Histogram of the non-fluorescent control shown in Fig. 2B.

Measurement of the fluorescence distribution of the non-fluorescent control, plotted as a histogram, and compared to the data shown in Fig. 2B.

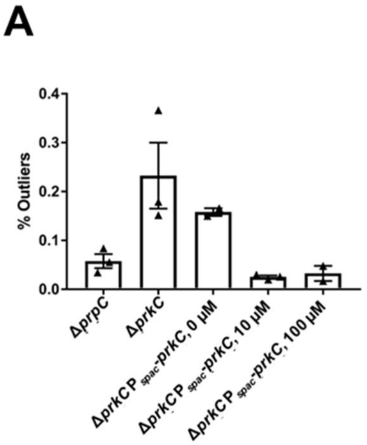

Genotype	% *sasA* outliers
**Δ*prpC***	0.058 ± 0.014
**Δ*prkC***	0.23 ± 0.067
**Δ*prkC* P_{spac}-*prkC* 0 µM**	0.16 ± 0.008
**Δ*prkC* P_{spac}-*prkC* 10 µM**	0.026 ± 0.0029
**Δ*prkC* P_{spac}-*prkC* 100 µM**	0.033 ± 0.015

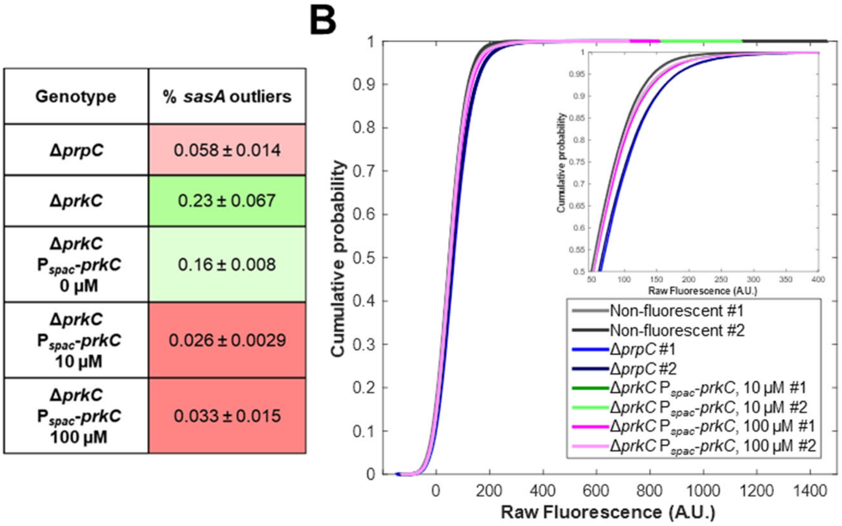

Supplementary Figure 6: Reduction of noise in a Δ*prkC* background by complementation.

A) Percentage of outliers in each genetic background. Similar experiments to those shown in Figure 2 were performed in *a* Δ*prkC* background with IPTG inducible *prkC* expression*.* Additional samples of Δ*prpC* and Δ*prkC* were included as controls. Each experiment was normalized to a control and outliers were defined as cells above a fixed threshold level of normalized fluorescence as in Figure 2. Triangles represent the percentage of each population (biological replicate) that is above the threshold; bars and lines represent the mean and SEM, respectively. Values are as listed in the table. (Samples were measured on a MACSquant flow cytometer – see materials and methods.)

B) Cumulative distribution functions of two biological replicates of the experiments shown in **A** compared to non-fluorescent controls. Note that fluorescence values shown are raw measurements (non-normalized). **Inset:** by the ~50th percentile (probability ~0.5), the populations with induced heterologous *prkC* expression show a systematic reduction in variability in *sasA* expression (shades of green and magenta) compared to Δ*prpC* (shades of blue) and approach the non-fluorescent control (shades of gray).

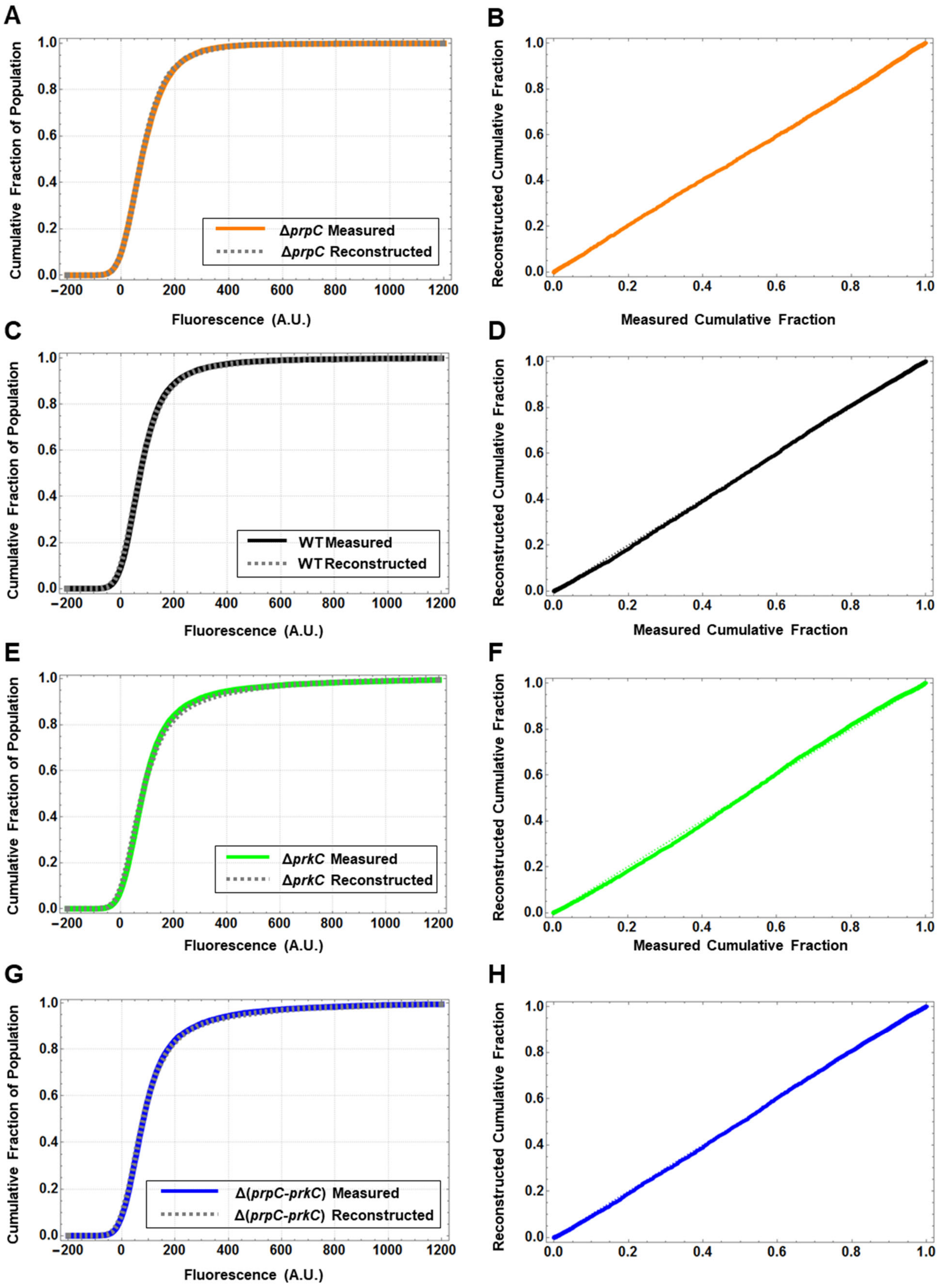
A
Cumulative Fraction of Population
Fluorescence (A.U.)
ΔprpC Measured
ΔprpC Reconstructed
B
Reconstructed Cumulative Fraction
Measured Cumulative Fraction
C
Cumulative Fraction of Population
Fluorescence (A.U.)
WT Measured
WT Reconstructed
D
Reconstructed Cumulative Fraction
Measured Cumulative Fraction
E
Cumulative Fraction of Population
Fluorescence (A.U.)
ΔprkC Measured
ΔprkC Reconstructed
F
Reconstructed Cumulative Fraction
Measured Cumulative Fraction
G
Cumulative Fraction of Population
Fluorescence (A.U.)
Δ(prpC-prkC) Measured
Δ(prpC-prkC) Reconstructed
H
Reconstructed Cumulative Fraction

Supplementary Figure 7: Validation of the autofluorescence deconvolution method used in Figure 2.

Comparison of the measured fluorescence distributions from Fig. 2B and reconstructed distributions based on the deconvolution algorithm (main text, methods). Plots of the cumulative distribution functions of the measured data sets (solid lines) and the data set numerically reconstructed (dashed lines) using the deconvolved data and the measured autofluorescence. This was repeated for each genotype **(A)** Δ*prpC*, **(C)** WT, **(E)** Δ*prkC*, **(G)** Δ(*prpC-prkC*). To assess the agreement between the measured and reconstructed data sets for each, P-P plots were also used. For each data set the measured cumulative fraction was plotted against the reconstructed cumulative fraction (solid). A line with a slope of 1 (dashed), indicates perfect agreement. This process was repeated for **(B)** Δ*prpC*, **(D)** WT, **(F)** Δ*prkC*, and **(H)** Δ(*prpC-prkC*), and the presence of only very small deviations indicates very good agreement between the reconstructed and measured data sets.

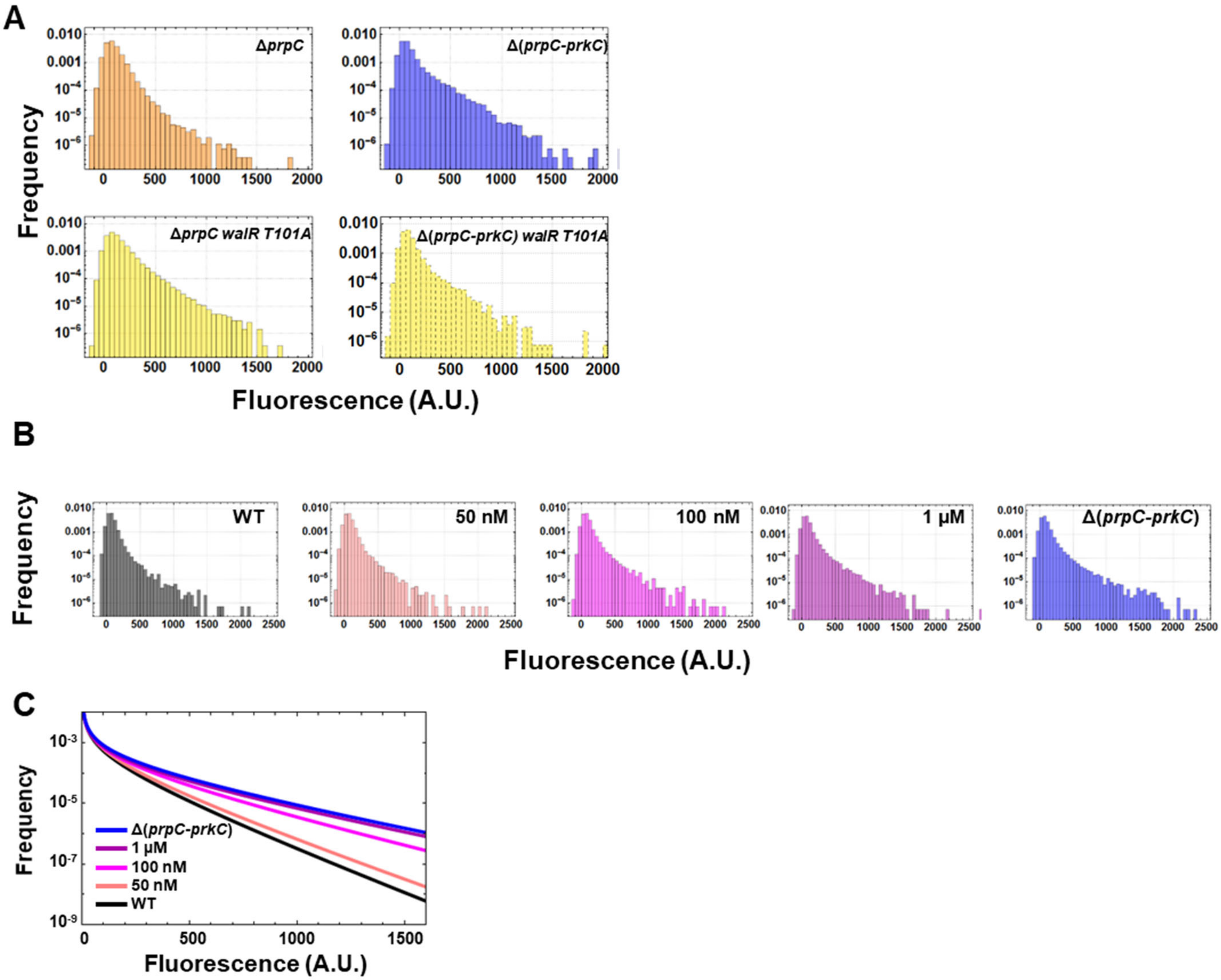

Supplementary Figure 8: Additional data sets used to generate Figure 3.

A) **Histograms of measured data used to generate Figure 3C.** P_{sasA}-*yfp* reporter activity was quantified by flow cytometry in a Δ*prpC walR T101A* (yellow, solid) background and compared to Δ*prpC* (orange), Δ(*prpC-prkC*) (blue), and Δ(*prpC-prkC*) *walR*^{T101A} (yellow, dashed) backgrounds in the same experiment. Each data set was obtained from ~6.0*10^4 events.

B) **Histograms of measured data used to generate Figure 3D.** The effect of staurosporine on *sasA* expression is dose-dependent. P_{sasA}-*yfp* reporter activity was

quantified by flow cytometry during treatment with increasing concentrations of staurosporine: 0 (solvent only; black), 50 nM, 100 nM, and 1 μM (shades of magenta), in otherwise WT populations. For reference, the distribution of P_{sasA}-*yfp* in a Δ(*prpC-prkC*) (blue) population treated with solvent only was also measured in the same experiment. Each distribution was measured from data on ~$3.0*10^4$ events.

C) Functional fits of autofluorescence-free distributions of *sasA* expression with increasing concentrations of staurosporine. A deconvolution algorithm was used to remove the contribution of autofluorescence from the measured distributions of *sasA* expression shown in **B**.

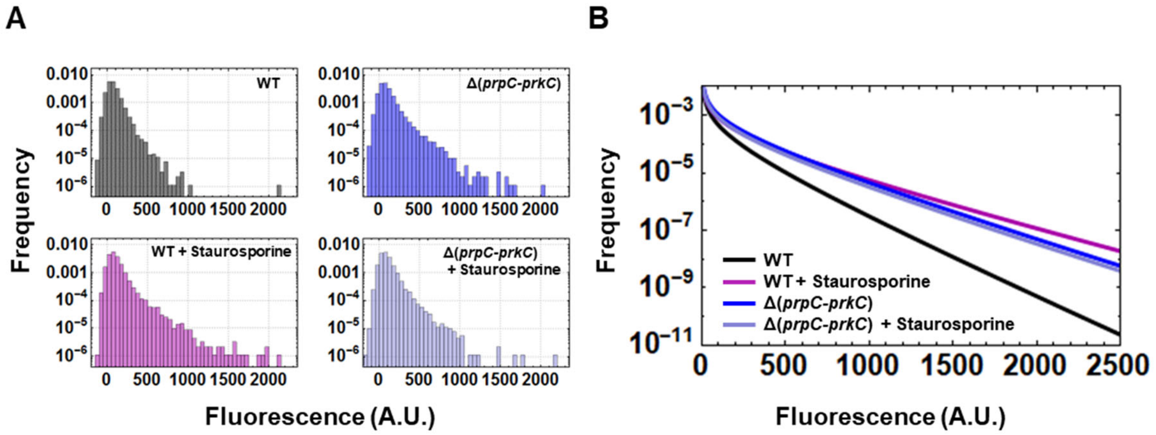

Supplementary Figure 9: Additional controls for the PrkC inhibition experiment shown in Fig 3.

A) **The effect of staurosporine on *sasA* requires the PrkC/PrpC system.** Treatment with the kinase inhibitor staurosporine results in a PrkC-dependent increase in the frequency of cells with elevated *sasA* expression. P_{sasA}-*yfp* reporter activity was quantified by flow cytometry in WT (black) or Δ(*prpC-prkC*) (blue) untreated populations, and WT (magenta) and Δ(*prpC-prkC*) (light blue) treated populations treated with 1 μM staurosporine. Each distribution was measured from data on ~3.0*10^4 events.

B) **Deconvolution of data shown in A from cellular autofluorescence.**

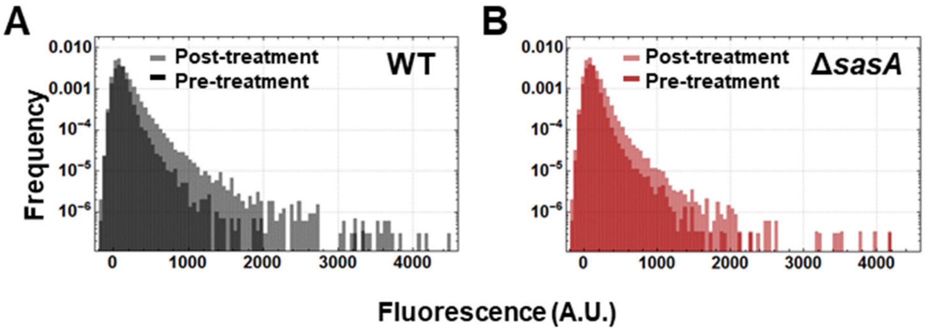

Supplementary Figure 10: Distributions of *sasA* expression pre- and post-ciprofloxacin treatment used to generate the fits and model in Fig. 4.

A) Antibiotic treatment results in an increase in the number of cells with elevated *sasA* expression. Distributions of P_{sasA}-*yfp* expression in an otherwise wild type background before (dark gray) and after (light gray) ciprofloxacin treatment. Data represents ~10^5 events for each population.

B) Histograms of the distributions of P_{sasA}-*yfp* expression in a Δ*sasA* background before (dark red) and after (light red) ciprofloxacin treatment in a parallel experiment to **A**.

A

Genotype	Survival @ 3h30min (mean ± range, 2 experiments)
WT	1.9 ± 0.1%
Δ*sasA*	2.5 ± 0.5%
Δ(*prpC-prkC*)	2.9 ± 1.5%

B

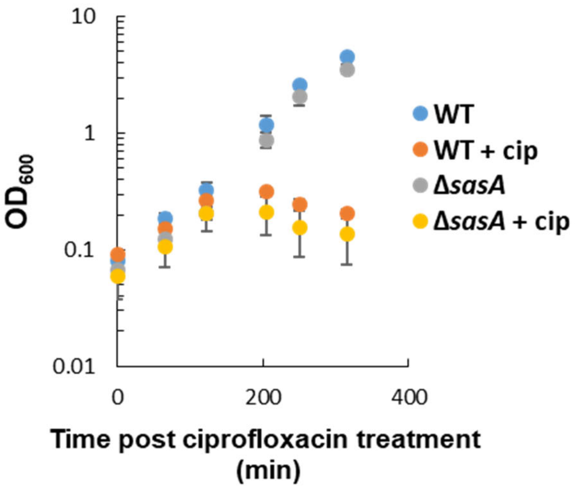

C

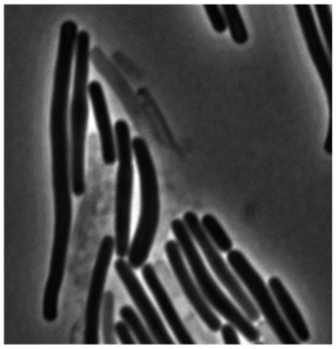

Supplementary Figure 11: Ciprofloxacin treatment of *B. subtilis* populations results in killing and lysis.

A) 3h 30 min of ciprofloxacin treatment results in ~1-2% survival of WT, Δ*sasA*, Δ(*prpC-prkC*) populations as assayed by colony forming units.

B) OD_{600} of ciprofloxacin treated cultures over time. Dots and lines indicate the means and ranges, respectively.

C) Cells sampled from a WT culture after 3.5h of ciprofloxacin treatment. Cultures were spun down gently to concentrate cells. Significant lysis is observed. Note that centrifugation is not expected to recover all debris.

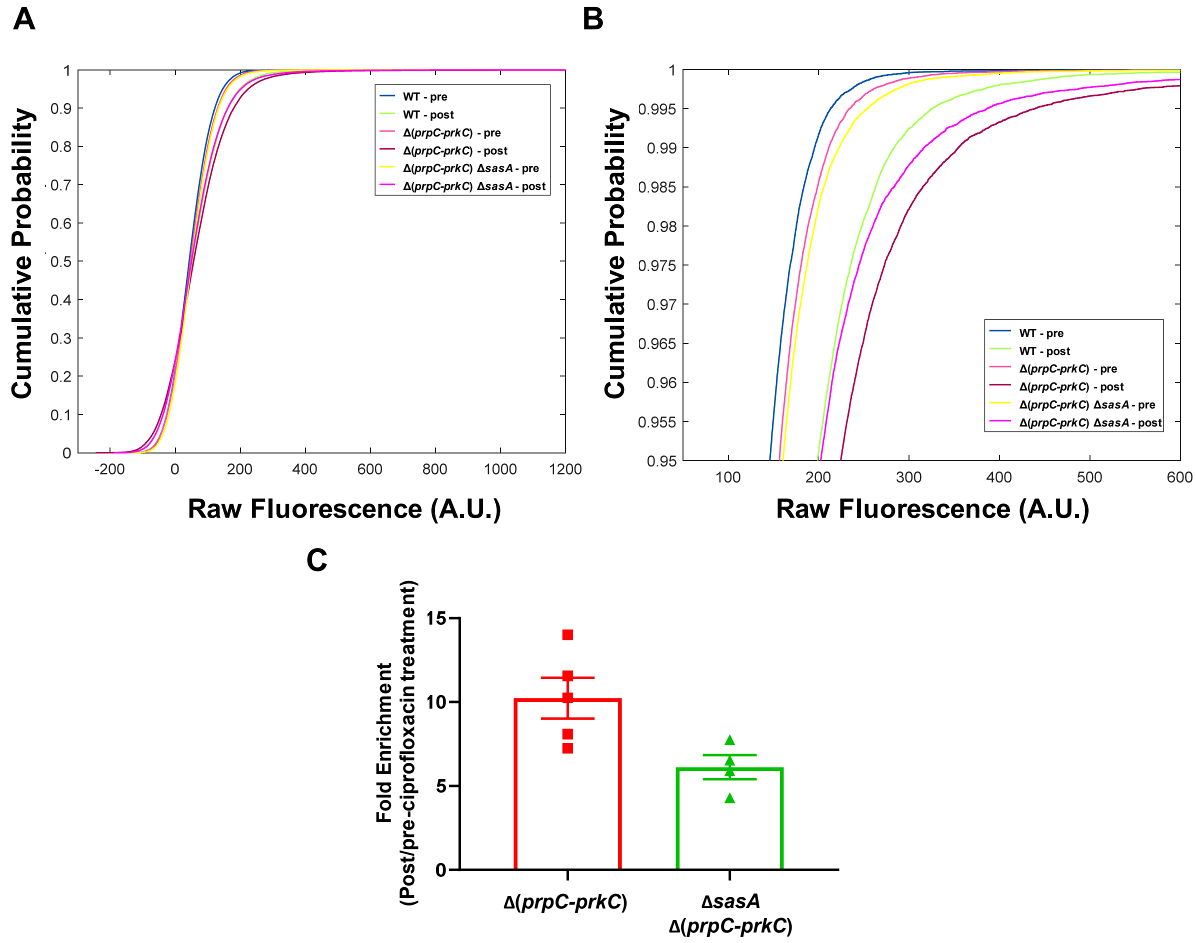

Supplementary Figure 12: Ciprofloxacin treatment enriches Δ(*prpC-prkC*) populations for cells with high levels of P_{sasA}.

A) Representative cdfs of YFP fluorescence of a P_{sasA}-*yfp* reporter in WT, Δ(*prpC-prkC*), and Δ(*prpC-prkC*) Δ*sasA* backgrounds pre- or post-ciprofloxacin treatment. (Samples were measured on a MACSquant flow cytometer – see materials and methods.)

B) detail of **A** above the 95th percentile. The frequency of cells with high levels of *sasA* expression that are the more tolerant than the bulk of the population (as in Fig. 4) is enriched by ~50% in Δ(*prpC-prkC*) populations compared to WT. (Compare WT-pre vs. Δ(*prpC-prkC*)-pre.) The frequency in Δ(*prpC-prkC*) populations post-treatment is similarly increased.

C) Fold enrichment of populations (biological replicates) at a fluorescence corresponding to ~99.5th percentile in the untreated populations (250 A.U.). The fold enrichment of Δ(*prpC-prkC*) is partially *sasA* mediated. (Two-tailed Mann-Whitney test, p-value ~ 0.03.) Bars and lines indicate the means and SEMs, respectively.

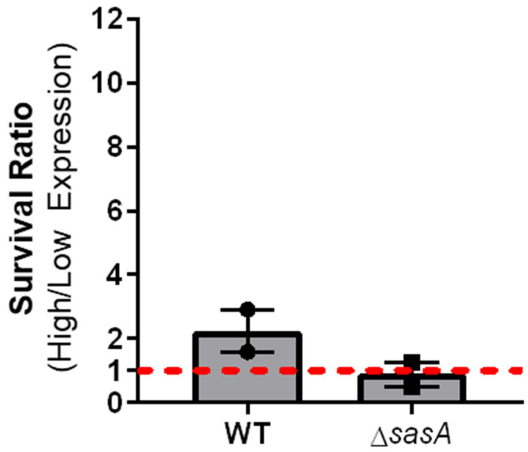

Supplementary Figure 13: Relaxing the "high" expression threshold results in a strongly reduced survival advantage. The "high" expression threshold is ~60% of the value of the threshold used in Fig. 4D,E. This results in a measured effect of ~ 2.3 ± 0.7 fold for WT, and 0.9 ± 0.4 fold for Δ*sasA* (mean ± range, 2 experiments), compared to a model prediction (Fig. 4C) of 3.7 and 2.0-fold, respectively. We concluded that size of the survival advantage at this threshold is not strong enough to report as a robust effect.

Supplementary Table 1. Strains in figures

Figure	Panel	Strains
**1**	A	ELB115
	B, C, D	ELB344
**2**	B	ELB115, ELB116, ELB117, ELB330
	C,D	ELB115, ELB116, ELB330, ELB371, ELB369B, ELB373
**3**	A,B	ELB115, ELB116, ELB117, ELB330
	C	ELB330, ELB116, ELB369B, ELB373
	D	ELB115, ELB330
**4**	A	ELB115
	B	ELB331
	C,D,E	ELB115, ELB331
**S1**	B,C	ELB499, ELB450
**S2**	A	ELB348, ELB452
	B,C,D	ELB348
**S3**	A,B,C	ELB205, ELB211, ELB217
	D,E,F	ELB243, ELB299, ELB249
**S4**	A	ELB80, ELB81, ELB82, ELB367
	B	ELB359, ELB360, ELB362, ELB374ELB349, ELB350, ELB351
	C,D	
**S5**		
**S6**	A,B	ELB116, ELB117, IP563
**S7**	A,B	ELB116
	C,D	ELB115
	E,F	ELB117
	G,H	ELB330
**S8**	A	ELB330, ELB116, ELB369B, ELB373
	B,C	ELB115, ELB330
**S9**	A,B	ELB115, ELB330
**S10**	A	ELB115
	B	ELB331
**S11**	A	ELB115, 330, 331
	B	ELB115, 331
	C	ELB115
**S12**	A,B	ELB115, ELB330, ELB357B
**S13**		ELB115, ELB331

Supplementary Table 2: Oligos used in this study

Name	Sequence (5'-3')
EcoRI-PywaC-u1	GGCTAGAATTCGTCCAGAACGAAATGCCGATG
BamHI-PywaC-l1	GGCTAGGATCCCGGAACTTTATCCGCTGTCC
pDG780-ywaC-u1	TTGGGTACCGGGCCCCCCCTCGAGGTCGACATTCAGACAGATAAGATCAATATG
Tet-ywaC-l1	ACAATATGGCCCGCTTTAACGGAACTTTATCCG
ywaC-tet-u1	AGTTCCGTTAAAGCGGGCCATATTGTTGTATAAG
ywaC-tet-l1	TTTCTCATCTAGGGAACTCTCTCCCAAAGTTG
Tet-ywaC-u1	TGGGAGAGAGTTCCCTAGATGAGAAAATGCTGG
pDG780-ywaC-l1	ACTAGTGGATCCCCCGGGCTGCAGGAATTCGTGAACTTCAACTTAGATATGGTAG
HindIII-mCherry-u1	GGTCAAAGCTTAAAGGAGGAAAGTCACATTATGGTTTCCAAGGGCGAGG
BamHI-mCherry-l1	GGTCAGGATCCTTATTTGTACAGCTCATCC
HindIII-PywaC-l2	GGTCAAAGCTTCGGAACTTTATCCGCTGTCC
EcoRI-PsigM-u1	GGCTAGAATTCCACTATCTTTTCCCCTCTGG
HindIII-PsigM-l1	GGTCAAAGCTTCTATGTTATACACGCATAAG
BamHI-PsigM-l1	GGCTAGGATCCCTATGTTATACACGCATAAG
IP_P_1130	AGCTCATTTATTGTACAACACGAGCCCATTTTTGTCAAATAAAATTTAAATTATATC AACGTTAATAAGG
IP_P_1131	AATTCCTTATTAACGTTGATATAATTTAAATTTTATTTGACAAAAATGGGCTCGTGT TGTACAATAAATG

Supplementary References

1 Gaidenko, T. A., Kim, T. J. & Price, C. W. The PrpC serine-threonine phosphatase and PrkC kinase have opposing physiological roles in stationary-phase Bacillus subtilis cells. *J Bacteriol* **184**, 6109-6114, doi:10.1128/jb.184.22.6109-6114.2002 (2002).

2 D'Elia, M. A. *et al.* Probing teichoic acid genetics with bioactive molecules reveals new interactions among diverse processes in bacterial cell wall biogenesis. *Chemistry & biology* **16**, 548-556, doi:10.1016/j.chembiol.2009.04.009 (2009).

3 Cao, M. & Helmann, J. D. Regulation of the Bacillus subtilis bcrC bacitracin resistance gene by two extracytoplasmic function sigma factors. *J Bacteriol* **184**, 6123-6129, doi:10.1128/jb.184.22.6123-6129.2002 (2002).

4 Libby, E. A., Goss, L. A. & Dworkin, J. The Eukaryotic-Like Ser/Thr Kinase PrkC Regulates the Essential WalRK Two-Component System in Bacillus subtilis. *PLoS Genet* **11**, e1005275, doi:10.1371/journal.pgen.1005275 (2015).

5 Eldar, A. *et al.* Partial penetrance facilitates developmental evolution in bacteria. *Nature* **460**, 510-514, doi:10.1038/nature08150 (2009).

6 Guerout-Fleury, A. M., Shazand, K., Frandsen, N. & Stragier, P. Antibiotic-resistance cassettes for Bacillus subtilis. *Gene* **167**, 335-336, doi:10.1016/0378-1119(95)00652-4 (1995).

7 Middleton, R. & Hofmeister, A. New shuttle vectors for ectopic insertion of genes into Bacillus subtilis. *Plasmid* **51**, 238-245, doi:10.1016/j.plasmid.2004.01.006 (2004).
