# Supplementary material for: Multisite phosphorylation drives phenotypic variation in (p)ppGpp synthetase-dependent antibiotic tolerance
Source: Nat Commun. 2019 Nov 13;10:5133. doi: 10.1038/s41467-019-13127-z (PMC6853874; doi:10.1038/s41467-019-13127-z)
Supplement: Supplementary file 3 — Description of Additional Supplementary Files [file 41467_2019_13127_MOESM3_ESM.pdf]

Description of Additional Supplementary Files

File Name: Supplementary Data 1

Description: Strains used in this study

File Name: Supplementary Data 2

Description: Plasmids used in this study
