# Supplementary material for: Multisite phosphorylation drives phenotypic variation in (p)ppGpp synthetase-dependent antibiotic tolerance
Source: Nat Commun. 2019 Nov 13;10:5133. doi: 10.1038/s41467-019-13127-z (PMC6853874; doi:10.1038/s41467-019-13127-z)
Supplement: Supplementary file 6 — Reporting Summary [file 41467_2019_13127_MOESM6_ESM.pdf]

nature research

Corresponding author(s): Jonathan Dworkin



# Reporting Summary

Nature Research wishes to improve the reproducibility of the work that we publish. This form provides structure for consistency and transparency in reporting. For further information on Nature Research policies, see Authors & Referees and the Editorial Policy Checklist.

## Statistical parameters

When statistical analyses are reported, confirm that the following items are present in the relevant location (e.g. figure legend, table legend, main text, or Methods section).

| n/a | Confirmed | |
|---|---|---|
| ☐ | ☒ | The exact sample size (*n*) for each experimental group/condition, given as a discrete number and unit of measurement |
| ☐ | ☒ | An indication of whether measurements were taken from distinct samples or whether the same sample was measured repeatedly |
| ☐ | ☒ | The statistical test(s) used AND whether they are one- or two-sided<br>*Only common tests should be described solely by name; describe more complex techniques in the Methods section.* |
| ☐ | ☒ | A description of all covariates tested |
| ☒ | ☐ | A description of any assumptions or corrections, such as tests of normality and adjustment for multiple comparisons |
| ☐ | ☒ | A full description of the statistics including central tendency (e.g. means) or other basic estimates (e.g. regression coefficient) AND variation (e.g. standard deviation) or associated estimates of uncertainty (e.g. confidence intervals) |
| ☐ | ☒ | For null hypothesis testing, the test statistic (e.g. *F*, *t*, *r*) with confidence intervals, effect sizes, degrees of freedom and *P* value noted<br>*Give P values as exact values whenever suitable.* |
| ☒ | ☐ | For Bayesian analysis, information on the choice of priors and Markov chain Monte Carlo settings |
| ☒ | ☐ | For hierarchical and complex designs, identification of the appropriate level for tests and full reporting of outcomes |
| ☐ | ☒ | Estimates of effect sizes (e.g. Cohen's *d*, Pearson's *r*), indicating how they were calculated |
| ☐ | ☒ | Clearly defined error bars<br>*State explicitly what error bars represent (e.g. SD, SE, CI)* |

*Our web collection on statistics for biologists may be useful.*

## Software and code

Policy information about availability of computer code

| | |
|---|---|
| Data collection | Nikon Elements AR (microscopy), BD FACSDiva (flow cytometry), MACSQuant VYB (flow cytometry) |
| Data analysis | Data was analyzed using Flowjo (flow cytometry), Fiji (microscopy), Matlab (microscopy & flow cytometry), and Excel (plate reader). |

For manuscripts utilizing custom algorithms or software that are central to the research but not yet described in published literature, software must be made available to editors/reviewers upon request. We strongly encourage code deposition in a community repository (e.g. GitHub). See the Nature Research guidelines for submitting code & software for further information.

## Data

Policy information about availability of data

All manuscripts must include a data availability statement. This statement should provide the following information, where applicable:

- Accession codes, unique identifiers, or web links for publicly available datasets
- A list of figures that have associated raw data
- A description of any restrictions on data availability

Primary data sets are available from the corresponding author upon request.

# Field-specific reporting

Please select the best fit for your research. If you are not sure, read the appropriate sections before making your selection.

☒ Life sciences ☐ Behavioural & social sciences ☐ Ecological, evolutionary & environmental sciences

For a reference copy of the document with all sections, see nature.com/authors/policies/ReportingSummary-flat.pdf

# Life sciences study design

All studies must disclose on these points even when the disclosure is negative.

| | |
|---|---|
| Sample size | Sample sizes were chosen for experiments testing low frequency events could be performed. e.g. by flow cytometry. For example, the number of cells in each experimental replicate was chosen to be at least 30k so that events present at ~1% would have roughly ~300 cells +/- 17 due to sampling. |
| Data exclusions | No data was excluded. |
| Replication | Experiments were replicated as indicated. Generally the spread between experiments was low, however outliers did occur. Experimental replicates with significant spread are included and indicated. |
| Randomization | Samples were not randomized, however experiments included controls for reference signals. Where possible samples were treated identically. |
| Blinding | Blinding was not performed. However, cell sorting experiments were performed by another individual (core manager) who was blinded to sample identity. |

# Reporting for specific materials, systems and methods

## Materials & experimental systems

| n/a | Involved in the study | |
|---|---|---|
| ☐ | ☒ | Unique biological materials |
| ☒ | ☐ | Antibodies |
| ☒ | ☐ | Eukaryotic cell lines |
| ☒ | ☐ | Palaeontology |
| ☒ | ☐ | Animals and other organisms |
| ☒ | ☐ | Human research participants |

## Methods

| n/a | Involved in the study | |
|---|---|---|
| ☒ | ☐ | ChIP-seq |
| ☐ | ☒ | Flow cytometry |
| ☒ | ☐ | MRI-based neuroimaging |

## Unique biological materials

Policy information about availability of materials

| | |
|---|---|
| Obtaining unique materials | All strains and plasmids generated for this study are available upon request. |

## Flow Cytometry

### Plots

Confirm that:

☒ The axis labels state the marker and fluorochrome used (e.g. CD4-FITC).

☒ The axis scales are clearly visible. Include numbers along axes only for bottom left plot of group (a 'group' is an analysis of identical markers).

☒ All plots are contour plots with outliers or pseudocolor plots.

☒ A numerical value for number of cells or percentage (with statistics) is provided.

### Methodology

| | |
|---|---|
| Sample preparation | As described in the methods: "Cultures were grown to early log phase and diluted 2-4 fold with additional S7 media to obtain the |

| | |
|---|---|
| Sample preparation | optimal density for flow cytometry. The resulting samples were vortexed vigorously prior to measurement to disrupt aggregates. " |
| Instrument | As described in the methods: Flow cytometry and sorting were performed on a BD Biosciences FACS Aria II-SORP or a Miltenyi MACSquant VYB (Figure S5 only). YFP was detected using a blue laser (488 nm) with a 525/50 dichroic, and a 505 long pass filter for both flow cytometers. mCherry was detected using a yellow/green laser (561 nm) with a 582/15 dichroic, and a 570 long pass filter (BD Aria only). |
| Software | As described in the methods:<br>Data collection: BD FACSDiva and MACSQuant VYB<br>Data analysis: Flowjo and Matlab |
| Cell population abundance | N/A. |
| Gating strategy | Cells were only gated to exclude debris/noise. Consistent gating was applied across all experiments. |

☐ Tick this box to confirm that a figure exemplifying the gating strategy is provided in the Supplementary Information.
